# Crystal structures of cholera toxin in complex with fucosylated receptors point to importance of secondary binding site

**DOI:** 10.1038/s41598-019-48579-2

**Published:** 2019-08-22

**Authors:** Joel B. Heim, Vesna Hodnik, Julie E. Heggelund, Gregor Anderluh, Ute Krengel

**Affiliations:** 1 Department of Chemistry, University of Oslo, P.O. Box 1033, NO-0315 Blindern, Norway; 2 Department of Biology, Biotechnical Faculty, University of Ljubljana, Jamnikarjeva 101, 1000 Ljubljana, Slovenia; 3 Department of Molecular Biology and Nanobiotechnology, The National Institute of Chemistry, Hajdrihova 19, 1000 Ljubljana, Slovenia; 4 Present Address: Lek d.d., Kolodvorska 27, 1234 Mengeš, Slovenia

**Keywords:** X-ray crystallography, Glycobiology

## Abstract

Cholera is a life-threatening diarrhoeal disease caused by the human pathogen *Vibrio cholerae*. Infection occurs after ingestion of the bacteria, which colonize the human small intestine and secrete their major virulence factor – the cholera toxin (CT). The GM1 ganglioside is considered the primary receptor of the CT, but recent studies suggest that also fucosylated receptors such as histo-blood group antigens are important for cellular uptake and toxicity. Recently, a special focus has been on the histo-blood group antigen Lewis^x^ (Le^x^), however, where and how the CT binds to Le^x^ remains unclear. Here we report the high-resolution crystal structure (1.5 Å) of the receptor-binding B-subunits of the CT bound to the Le^x^ trisaccharide, and complementary quantitative binding data for CT holotoxins. Le^x^, and also l-fucose alone, bind to the secondary binding site of the toxin, distinct from the GM1 binding site. In contrast, fucosyl-GM1 mainly binds to the primary binding site due to high-affinity interactions of its GM1 core. Le^x^ is the first histo-blood group antigen of non-secretor phenotype structurally investigated in complex with CT. Together with the quantitative binding data, this allows unique insight into why individuals with non-secretor phenotype are more prone to severe cholera than so-called ‘secretors’.

## Introduction

Cholera is an acute and severe diarrhoeal disease that, if left untreated, can cause serious dehydration and death within hours^1^. The disease is a major burden, especially where proper sanitation is lacking, in war zones and after natural disasters. The socio-economic costs of cholera are significant, and a global road map to end cholera has been established^2^. The current outbreak in Yemen is the worst in recent history with more than one million reported cases^3,4^, further aggravating the ongoing malnutrition crisis.

**Figure 1:**
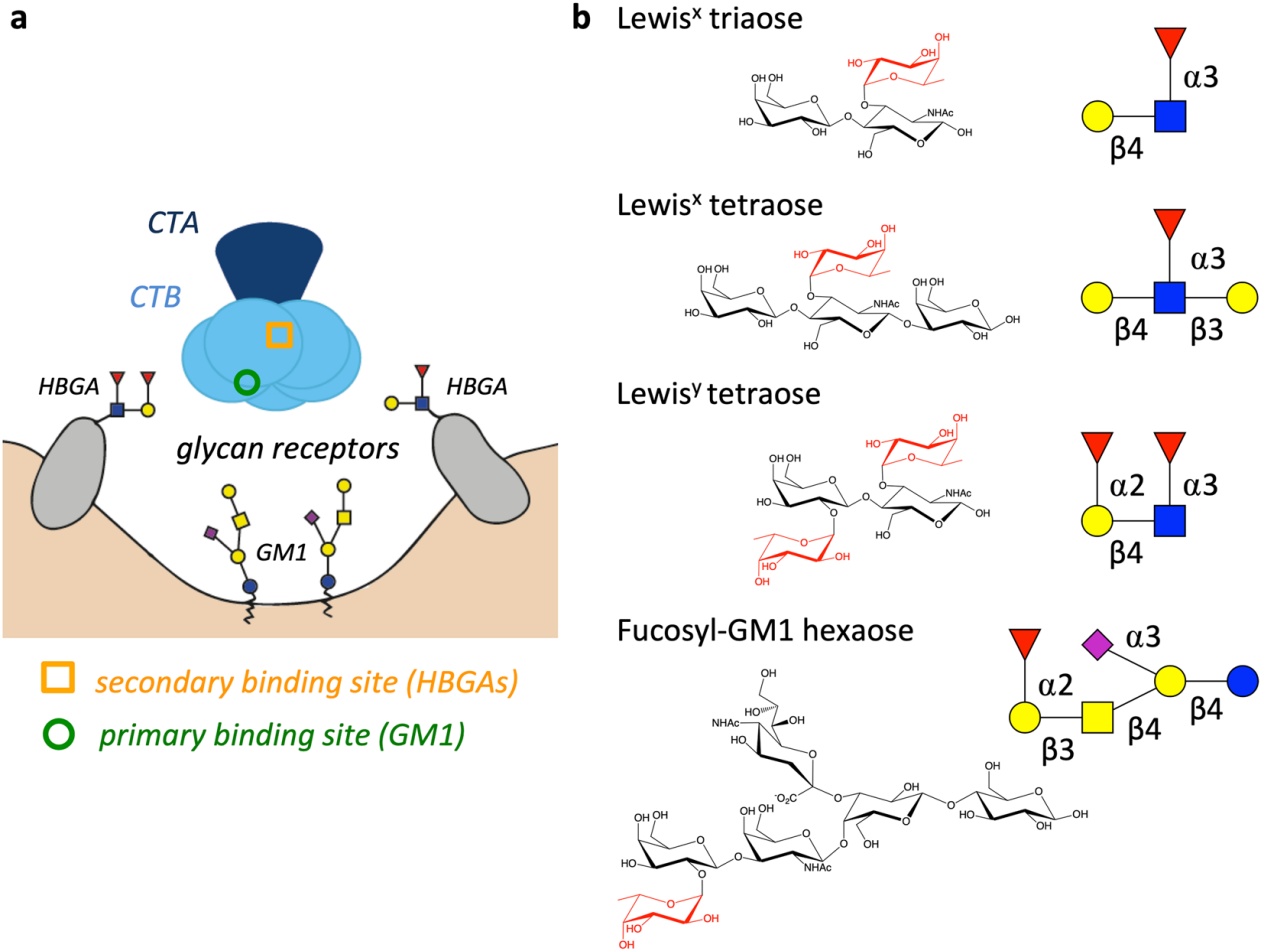
Schematic drawing of CT and its glycan receptors. (**a**) Toxin binding to host cell. The AB_5_ toxin CT consists of one catalytically active A subunit (CTA; dark blue) and a pentamer of the receptor-binding B subunits (CTB; light blue). The toxin’s primary and secondary binding sites (for GM1 and HBGAs, respectively) are indicated on one of the five B subunits with a green circle and an orange rectangle, respectively. (**b**) Structures of the oligosaccharides Lewis^x^ triaose and tetraose, Lewis^y^ tetraose and fucosyl-GM1 hexaose. Fucose residues are highlighted in red. Carbohydrate symbols follow the nomenclature of the Consortium for Functional Glycomics (Nomenclature Committee, Consortium for Functional Glycomics; d-galactose (Gal)–yellow circle, *N*-acetylgalactosamine (GalNAc)–yellow square, d-glucose (Glc)–blue circle, *N*-acetylglucosamine (GlcNAc)–blue square, l-fucose (Fuc)–red triangle*, N*-acetylneuraminic acid (Neu5Ac)–purple filled diamond.

Cholera is easily transmitted between humans through the faecal-oral route, *e*.*g*. by consuming food or water contaminated with the bacterium *Vibrio cholerae*. The bacteria colonize the small intestine and secrete the cholera toxin (CT)^5,6^. CT belongs to the protein family of AB_5_ toxins, which comprise one catalytically active A-subunit and a homopentamer of non-toxic B-subunits^6,7^ (Fig. 1a). The CT B-pentamer (CTB) facilitates binding to epithelial cells in the small intestine and subsequent cellular uptake of the holotoxin^6,8^. Once in the cytosol, the A-subunit hijacks the cells’ own signalling pathways, causing the secretion of chloride ions into the intestinal lumen and, due to the osmotic gradient, ultimately the severe watery diarrhoea typical of cholera^8–10^.

Key aspects of cholera intoxication are still poorly understood, starting with the interplay between the toxins and their cellular receptors, and the cellular uptake mechanism. For decades, the GM1 ganglioside has been considered to be the only cellular receptor for the CT^11–13^. The binding of CT to the branched GM1 pentasaccharide is one of the strongest protein-carbohydrate interactions known (*K*_d_ = 43 nM)^14^. Target recognition is mediated mainly by the two terminal sugar residues galactose and sialic acid, *via* a “two-fingered grip”^15^. A large body of research points towards this high-affinity interaction being the main entryway for the CT^12,16–20^. For example, the incorporation of exogenously added GM1 in human intestinal mucosal cells or rabbit small bowel segments lead to a concentration-dependent increase in the number of toxin-binding structures and intoxication^12,16^. CT binds GM1 at the “bottom” surface of the B-pentamer, which serves as a landing platform on the cell membrane^15^ (Fig. 1a). More recently, CT was found to also bind histo-blood group antigens (HBGAs), at a secondary binding site on the lateral side of the B-pentamer^21^. There is now increasing evidence that GM1 is not the only CT receptor and that also fucosylated structures like HBGAs play an important role for toxin binding, internalization and toxicity^13,21–34^.

HBGAs are present on the surface of cells in the gastrointestinal tract as well as in bodily secretions, and their expression varies dependent on the individual’s genotype. Intriguingly, CT causes blood-group-dependent cellular intoxication^31^ despite having only low affinity for human HBGAs, with a *K*_d_ in the millimolar range^21,27–29^. Blood group H determinants, which are characteristic for blood group O, exhibit slightly higher affinity to CT than blood group A and B determinants (still in the millimolar range); and patients with blood group O are the most likely to get severe cholera symptoms^35–38^. Another group with increased risk of severe cholera are people with non-secretor phenotype. These individuals have a non-functional *Se* (*FUT2*) gene, resulting in reduced fucosylation and the absence of HBGAs from bodily secretions^39^. Compared to GM1, HBGAs are present in the intestine in high concentrations^40^, making them attractive targets for toxin entry. Indeed, Le^x^, a monofucosylated HBGA expressed on human granulocytes and intestinal cells, has been shown to specifically bind to CTB and was proposed to function as a cellular receptor^32^. Furthermore, cholera intoxication in mice can be independent of GM1^32^, and fucose-based inhibitors are potent inhibitors of CT binding and internalization of intestinal cells^33,34^. It is, however, not clear how the CT binds to Le^x^ and related structures. Recent experiments with CT variants and structure-activity relationship data were interpreted such that Le^x^ binds to the primary binding site^32^. However, we find this unlikely since all CT-related crystal structures with HBGAs or HBGA-analogues to date feature these ligands in the secondary binding site^21,25,26^.

We hypothesized that Le^x^ and related sugars interact solely with the secondary binding site of CTB based on their similarity to known toxin-bound HBGAs and human milk oligosaccharides (HMOs)^21^. The only fucosylated ligand that we deemed likely to bind to the primary binding site was fucosyl-GM1, due to its similarity with GM1. To test this hypothesis, we investigated the binding of various fucosylated structures, including the fucosyl-GM1 oligosaccharide (os), to CTB and CTB variants by X-ray crystallography and surface plasmon resonance (SPR) spectroscopy.

## Results

To characterize the interaction of CT with different fucosylated ligands, we co-crystallized its receptor binding B subunits (classical CT biotype) with the Le^x^ trisaccharide, l-fucose and the fucosyl-GM1 hexasaccharide (fucosyl-GM1os) (Fig. 1b). The crystal structures were solved to high resolution (1.5–2.0 Å), based on high-quality X-ray data sets (Table 1). As expected, Le^x^ and l-fucose occupied the secondary binding sites of the CT, while we found fucosyl-GM1os in the primary toxin binding site, binding the CT with the same two-fingered grip as GM1. Its fucose residue contributes little to this interaction, serving more as decoration. Additionally, some of the secondary binding sites showed weak electron density for fucosyl-GM1os. In this case, the interaction was mediated *via* fucose.

In addition to the qualitative characterization of the CT-ligand interactions by X-ray crystallography, we acquired quantitative binding data by SPR spectroscopy (Table 2). Le^x^ was found to bind more weakly to the CT than the difucosylated HBGA Le^y ^^21^, but still had an affinity in the millimolar range, as typical for many protein-carbohydrate interactions. These studies were done with CTB as well as with the CT holotoxin. We also tested several CT variants with mutations in the primary or secondary binding sites. Specifically, we chose CT mutant W88K to knock out the primary binding site, since Trp88 serves an important role for aromatic stacking interactions with the terminal galactose residue in GM1. In line with the crystallographic data, primary binding site knock-out hardly affected CT binding to Le^x^. In contrast, a mutation in the secondary binding site (H18A) resulted in reduced binding to Le^x^. Intriguingly, this toxin variant not only bound more weakly to Le^x^, but concomitantly showed enhanced binding to GM1os, suggesting allosteric cross-talk between the two binding sites.

In the following, we give further technical details of our results.

### Crystal structures

#### CTB complex with Le^x^

**Figure 2:**
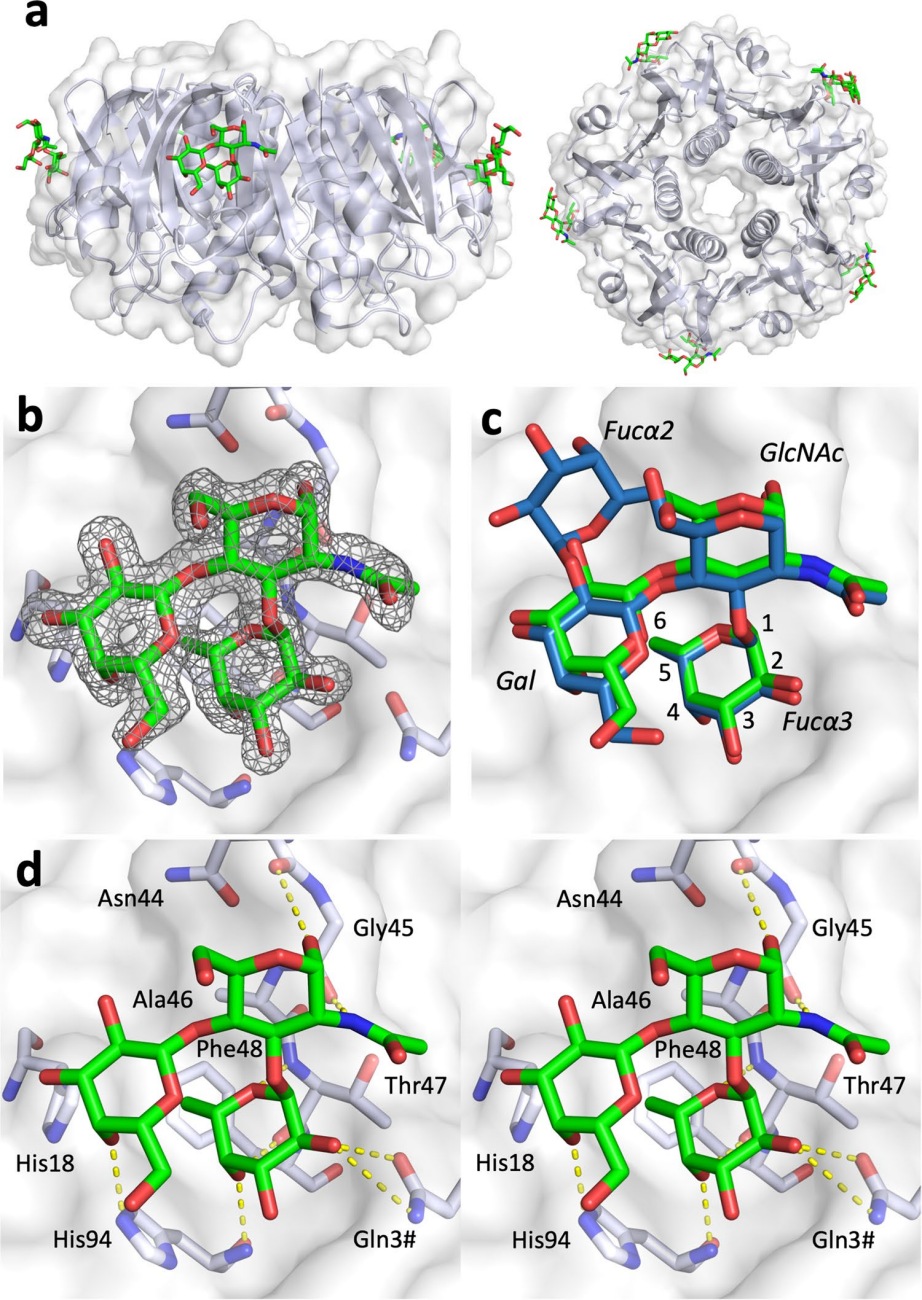
Lewis^x^ binds to the secondary binding site of the cholera toxin. (**a**) X-ray structure of CTB in complex with Le^x^ (PDB ID: 6HJD, this work); side and top views (rotated by 90°). The toxin B-pentamer is shown in cartoon and transparent surface representation, and the ligands depicted in stick representation. Le^x^ only binds to secondary binding sites on the lateral side of the toxin. (**b**) Close-up view of the secondary binding site (chain B), with σ_A_-weighted *F*_o_-*F*_c_ electron density map for Le^x^ (grey mesh, contoured at 3.0 σ, generated before placing the ligand) and selected residues depicted in green stick representation. (**c**) Superimposition of Le^y^ tetraose (blue sticks; PDB ID: 5ELB^21^) on CTB complex with Le^x^ triaose (green sticks; PDB ID: 6HJD, this work). Carbohydrate residues are labelled in italics, and fucose carbons are numbered. (**d**) Stereo image of the carbohydrate-toxin interactions. Hydrogen bonds are shown as yellow dashed lines, amino acid residues are labelled with 3-letter code. A hash (#) identifies residues contributed by a neighbouring subunit in the toxin B-pentamer. The figure was prepared with MacPyMol (Schrödinger LLC (www.schrodinger.com/pymol); version 1.8.0.3).

To study the interaction of CT with Le^x^, we crystallized purified CTB in complex with Le^x^ triaose. Crystals were obtained in two different crystallization conditions. They belonged to space group *P*2_1_2_1_2_1_ and contained two CTB pentamers in the asymmetric unit. Therefore, we can observe a total of 20 crystallographically distinct primary and secondary binding sites (ten of each). The protein structures show the typical “doughnut-shaped” CTB structure of five symmetrically arranged B subunits, each consisting of two three-stranded antiparallel β-sheets with α-helices on both sides^15^. The structures were determined to 2.0 Å and 1.5 Å resolution, respectively. In both crystal forms, Le^x^ is only observed in the secondary binding sites of the CT. The binding mode and interactions of Le^x^ described here are based on the 1.5 Å resolution structure, which was refined to a high-quality model (Fig. 2a, Table 1; PDB ID: 6HJD, *R*_free_ = 22.4%). Generally, the structure is well defined except for the flexible loop comprising residues 50–61 and the C-terminal asparagine residue, which exhibits some disorder. Inspection of the electron density maps revealed the presence of Le^x^ triaose in eight of ten secondary binding sites (Fig. 2a,b). One additional binding site contained electron density of sufficient quality to place the fucose residue, and the last secondary binding site was blocked by crystal contacts. No ligand density was observed in any of the primary binding sites, even at low sigma cut-off levels.

**Table 1:** Data collection and refinement statistics.

Protein	CTB + Lewis^x^	CTB + fucose	CTB + fucosyl-GM1os
**Data collection**			
Space group	*P*2_1_2_1_2_1_	*C2*	*C2*
**Cell dimensions**			
a, b, c (Å)	83.0, 108.3, 115.6	115.8, 100.5, 93.0	148.6, 74.1, 111.2
β (°)	90	117.0	105.6
Resolution (Å)	83.0–1.54* (1.57–1.54)**	47.4–1.95* (2.00–1.95)**	44.3–1.60* (1.63–1.60)**
No. of unique reflections	153937 (7547)	67464 (4577)	150674 (7491)
*CC*_(1/2)_ (%)	98.4 (53.5)	99.3 (41.1)	97.8 (44.9)
(I)/σ(I), *R*_merge_	8.9 (1.7), 0.11 (0.83)	6.2 (1.2), 0.12 (1.00)	4.9 (1.4), 0.14 (0.77)
Multiplicity	6.5 (6.4)	2.7 (2.6)	2.6 (2.6)
Completeness (%)	99.9 (99.5)	97.7 (98.5)	98.4 (98.7)
**Refinement**			
Resolution (Å)	83.0–1.54	47.4–1.95	44.32–1.60
*R*_cryst_/*R*_free_ (%)	19.3/22.4	18.7/23.7	18.4/21.4
No. of atoms			
Protein	8718	8218	8692
Ligand/ion	445/10	280/10	1380/10
Water	668	261	797
*B*-factors (Å^2^)			
Protein	18.9	31.8	13.3
Ligand /ion	24.0/15.2	34.0/25.6	18.4/10.9
Water	23.7	27.5	19.8
r.m.s.d. bonds (Å)	0.02	0.01	0.02
r.m.s.d. angles (°)	1.70	1.68	1.83
PDB ID	6HJD	6HMW	6HMY

*Data collected on a single crystal. **Values for the highest resolution shell are shown in parentheses.

Le^x^ triaose superimposes well with the trisaccharide core of previously characterized HBGA determinants (Fig. 2c), and engages in similar H-bonding interactions (Fig. 2d, Supplementary Table S1)^21^. Its reducing end *N*-acetylglucosamine (GlcNAc) points towards residue 47. The secondary binding site of CT recognizes HBGAs mainly through the α1,3-linked fucose (although Le^y^ can bind the CT in two orientations, where either Fucα2 or Fucα3 can serve as main anchoring points). As observed for CTB bound to Le^y^ and A-Le^y^ (5ELB^21^, 5ELD^21^), fucose forms hydrogen bonds to the side chain of Gln3# from the adjacent B-subunit and to the main chain of residues 47 and 94 (Fig. 2d, Supplementary Table S1). These data confirm our hypothesis that Le^x^ binds to the secondary CT binding site, similarly to other HBGAs.

#### CTB complex with l-fucose

**Figure 3:**
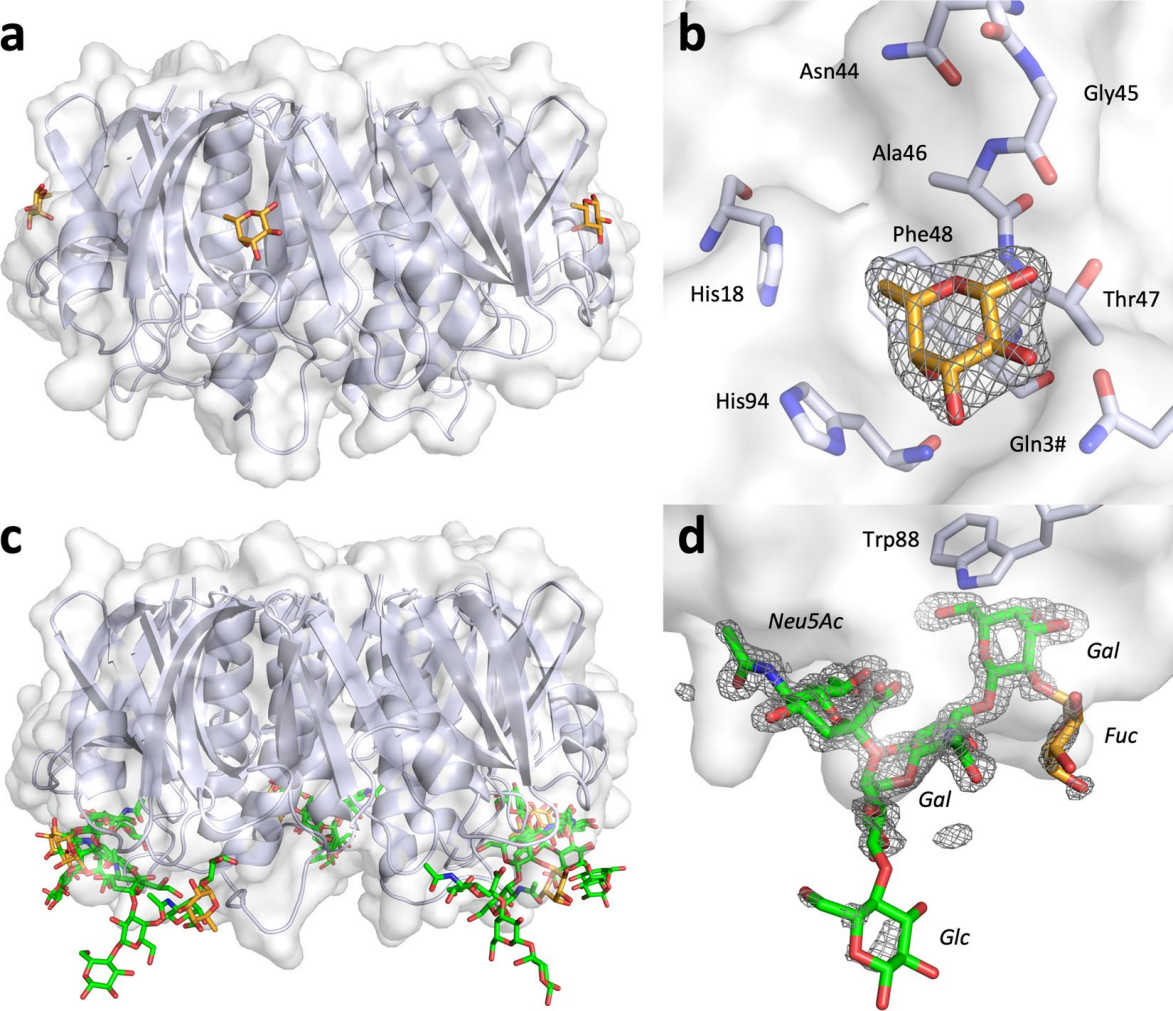
CTB complexes with l-fucose and fucosyl-GM1os. l-fucose binds to the secondary CT binding site, while fucosyl-GM1os binds to the primary binding site, facing the cell membrane. The cholera toxin B-pentamer is shown in cartoon and transparent surface representations, and the ligands depicted in stick representation. (**a**) X-ray structure of CTB in complex with l-fucose (orange sticks; PDB ID: 6HMW, this work); side view. (**b**) Close-up view of the secondary binding site, with σ_A_-weighted *F*_o_-*F*_c_ electron density map for l-fucose (grey mesh, contoured at 3.0 σ, generated before placing the ligand), and selected residues shown in stick representation, amino acid residues are labelled with 3-letter code. (**c**) X-ray structure of CTB in complex with fucosyl-GM1os (green sticks, with the terminal fucose highlighted in orange; PDB ID: 6HMY, this work). (**d**) Close-up view of the primary binding site, with σ_A_-weighted *F*_o_-*F*_c_ electron density map for fucosyl-GM1os (grey mesh, contoured at 3.0 σ, generated before placing the ligand) and Trp88 shown in stick representation. Carbohydrate residues are labelled in italics. The terminal fucose and glucose residues show weaker electron density compared to the four core residues of fucosyl-GM1os.

A recent study suggested that Le^x^ binds to the GM1 binding site^32^, however, all relevant toxin crystal structures contain fucosylated molecules in the secondary binding site^21,25^. To alleviate concerns that protein purification by galactose affinity chromatography may hinder access to the GM1 binding site due to the presence of residual galactose (even though Gal is not observed in our crystal structures), we produced CTB by TALON affinity chromatography exploiting its known affinity to Ni^2+^ or Co^2+^ ions^41^. To directly test the hypothesis by Cervin *et al*. that fucosylated sugars may bind to the GM1 binding site, TALON-purified CTB was co-crystallized with l-fucose in a molar ratio of 1:500 (B-subunit to ligand). The crystals belonged to space group *C2* and contained two CTB pentamers in the asymmetric unit. The structure was refined to 1.95 Å resolution and an *R*_free_ of 23.7% (Table 1). The electron density of the loop regions and the C-terminal Asn103 were more disordered compared to the CTB-Le^x^ structure, however, overall the structure is well defined. Electron density for β-l-fucose is observed in nine of ten secondary binding sites (Fig. 3a,b, Supplementary Table S2). We did not observe electron density corresponding to l-fucose in the GM1 binding site, confirming that fucose only binds to the secondary binding site. Additionally, l-fucose was found sandwiched between two B-pentamers and covalently attached to some of the N-terminal residues. The latter is likely due to non-enzymatic glycosylation^42^. These interactions are unlikely to be biologically relevant and probably caused by the high molar excess of l-fucose added before crystallization.

#### CTB complex with fucosyl-GM1os

Our data strongly suggest that the toxin secondary binding site is the sole binding site for Le^x^ and similar sugars. There is, however, one fucosylated oligosaccharide that would be expected to bind to the primary binding site, namely fucosyl-GM1os. Fucosyl-GM1 binds CT almost as strongly as GM1^43^. We crystallized TALON-purified CTB with fucosyl-GM1os in a 1:10 molar ratio (B-subunit to ligand), yielding crystals diffracting to 1.6 Å resolution. The structure was refined to an *R*_free_ of 21.4%. Inspection of the electron density revealed that fucosyl-GM1os indeed binds to the primary binding site, similarly to GM1os, with the additional fucose residue facing outwards toward the solvent (Fig. 3c). The electron density for the fucose residue is less well defined compared to the other sugar residues (Fig. 3d), suggesting that it binds more weakly. It also has higher *B*-factors (average *B*-factors for sugar ring atoms in chain A: Fuc > GalNAc > Gal, 28.6 > 20.8 > 17.1 Å^2^). This explains the small difference in CT affinity between fucosyl-GM1 and GM1, as the fucose residue is not a major contributor to the interaction. In addition to the primary binding sites, we observe fucose binding in some of the toxin’s secondary binding sites. In these sites, fucosyl-GM1os is not well resolved, however, electron density extending from the fucose is compatible with a larger oligosaccharide like fucosyl-GM1os.

### Protein interaction by SPR

#### CTB and Le^x^ triaose

To determine the binding affinity of CTB to Le^x^, we performed SPR experiments, in which the toxin B-pentamer was immobilized on the SPR chip and the sugar was injected as the analyte over a range of different concentrations. Le^x^ triaose was found to have a lower binding affinity to CTB than Le^y^ tetraose or A-Le^y^ pentaose, which both feature a second fucose residue (*K*_d_ = 10 ± 3 mM (Le^x^ triaose), compared to 1.05 ± 0.04 mM (Le^y^) and 2.2 ± 0.1 mM (A-Le^y^)^21^; Supplementary Fig. S1).

#### CT holotoxin and Le^x^ triaose and tetraose

**Figure 4:**
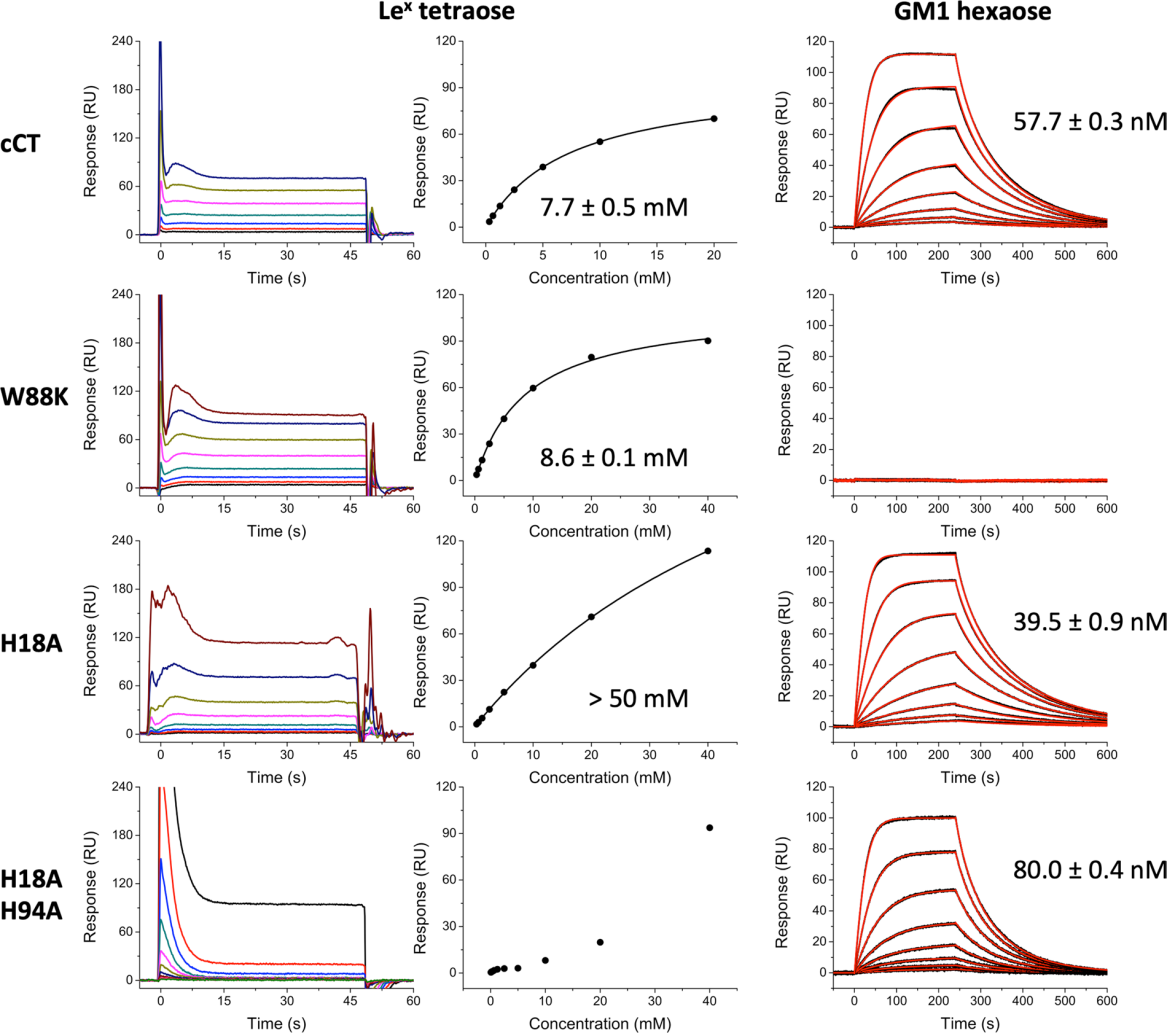
SPR sensorgrams and affinity plots for cholera toxin variants. SPR experiments were performed with CT holotoxins coupled to the sensor chip and using Le^x^ tetraose (sensorgrams and corresponding plots of steady state response against concentration) or GM1os (sensorgrams with model fit) as analytes, as indicated in the panel legends. *K*_d_ values were calculated from the steady state plots (Le^x^) or using a Langmuir 1:1 model (GM1os). The *K*_d_ value for H18A + Le^x^ could not be calculated since saturation was not reached upon addition of 40 mM ligand. H18AH94A only showed weak interaction for the highest Le^x^ concentrations used; therefore, the affinity plot does not show a saturation-binding curve.

Hitherto all crystal structures of CTB complexes with fucosylated receptors, including the structures reported here, suggest that Le^x^-related antigens bind exclusively to the secondary binding site of CT. To verify this binding mode in solution, we performed SPR experiments with holotoxin variants harbouring mutations in the primary binding site (W88K) or the HBGA binding site (H18A, H18AH94A; with residue 94 linking the primary and secondary binding sites) (Fig. 4, Table 2). Native folding of these variants was confirmed by circular dichroism (CD) spectroscopy (Supplementary Fig. S2). Toxin variants or wild-type CT were immobilized on SPR chips, before injecting oligosaccharides at different concentrations. First, we showed that substitution of W88A in the primary binding site prevented GM1os binding (Fig. 4). The other toxin variants bound GM1os similarly to wild-type CT, indicating intact primary binding sites. We then compared binding of Le^x^ oligosaccharides to wild-type CT and CT variants.

**Table 2:** *K*_d_ values for CTB and CT variants and glycans GM1os, Le^x^ triaose and tetraose determined by SPR. For comparison, CTB binds to Le^y^ tetraose with a *K*_d_ of 1.05 ± 0.04 mM^21^.

	GM1os	Le^x^ triaose	Le^x^ tetraose
CTB	not tested	10 ± 3 mM	not tested
CT	57.7 ± 0.3 nM	8 ± 3 mM	7.7 ± 0.5 mM
W88K	no binding	not tested	8.6 ± 0.1 mM
H18A	39.5 ± 0.9 nM	not tested	>50 mM
H18AH94A	80.0 ± 0.4 nM	not tested	very weak binding

Le^x^ triaose binds equally well to CTB and CT (*K*_d_ = 10 ± 3 mM for CTB, 8 ± 3 mM for CT) (Supplementary Fig. S1). This result was expected, since there is no major structural difference between free CTB and the B-pentamer in the holotoxin^15,44^. In our crystal structures, Le^x^ and related sugars pre-dominantly bind with the α-anomer of the reducing end GlcNAc, whereas relevant glycoconjugates on the cell surface are β-glycosidically linked to proteins or lipids. Le^x^ tetraose, with a fixed β-anomer, and Le^x^ triaose bound equally well to CT (*K*_d_ = 8.1 ± 3.2 mM for triaose, 7.7 ± 0.5 mM for tetraose), suggesting that the triaose core is mainly responsible for binding and that the linkage does not significantly affect binding affinity.

Next, we determined binding affinities for all toxin variants using Le^x^ tetraose (Fig. 4, Table 2). The primary binding site variant W88K was found to bind Le^x^ tetraose with an affinity comparable to wild-type CT (*K*_d_ = 8.6 ± 0.1 mM for W88K, 7.7 ± 0.5 mM for CT), whereas H18A, which features a mutation in the secondary binding site, exhibited significantly reduced binding to Le^x^ tetraose (*K*_d_ > 50 mM for H18A, 7.7 ± 0.5 mM for CT), in agreement with our structural data. This reduced binding affinity is not due to disruption of the primary binding site, since GM1os binding was found to be even stronger than for the wild-type protein (*K*_d_ = 39.5 ± 0.9 nM for H18A, 57.7 ± 0.3 nM for CT; Fig. 4). The double mutant H18AH94A, which was designed to disrupt the water network in the binding site and additionally precludes H-bond formation to the His94 side chain, only bound Le^x^ tetraose at the highest analyte concentration applied (40 mM), but bound to GM1os almost as strongly as wild-type CT (*K*_d_ = 80.0 ± 0.4 nM for H18AH94A, 57.7 ± 0.3 nM for CT). Our results confirm Le^x^ binding to the secondary binding site of CT and suggest cross-talk between the primary and secondary binding sites, since mutations in the latter affect GM1 binding, reducing the *K*_d_ by one third.

## Discussion

### Le^x^ and similar fucosylated structures bind to the secondary binding site of CT

We set out to explore the molecular interaction of CT with fucosylated receptors, in particular Le^x^. Already twenty years ago, CTB was shown to bind fucose^45^. More recently, evidence was presented that HBGAs may act as functional toxin receptors for the related LTB^46,47^, and fucosylated structures were shown to be functionally active and cause cellular uptake of CT^30,32–34^. For example, CTB binding to jejunal epithelial cells can be blocked by the Le^x^ trisaccharide or a monoclonal antibody against Le^x ^^32^. Crosslinking and immunoprecipitation of CTB-bound cellular proteins revealed that CTB binds to glycoproteins modified with Le^x ^^32^. It was, however, unclear how Le^x^ binds to CT. Since G33D, a primary site CTB variant with greatly reduced affinity to GM1^17,48^, showed weaker binding to Le^x ^^32^, it was suggested that also Le^x^ might bind to the primary binding site^32^. To explore this possibility, Cervin *et al*. performed competition experiments with plate-bound GM1 or Le^x^ and CTB pre-incubated with the free sugars^32^. Competition experiments were also performed with human granulocytes, T84 and Colo205 cells. Despite the fact that these cell lines only express low levels of GM1, pre-incubation of CTB with GM1os had an inhibitory effect. Taken together, these results were interpreted to indicate that GM1 competitively blocks fucosylated sugars from binding to the CT primary binding site^32^. However, it should be noted that even at high concentrations, GM1os could not block CTB binding completely and Le^x^ could not block binding to plate-bound GM1.

Here we show that Le^x^ binds solely to the secondary binding site of CTB, and even l-fucose alone (when co-crystallized in 500-fold molar excess) did not bind to the primary binding site. Moreover, SPR experiments with toxin variants confirmed that disruption of the secondary binding site, but not the primary binding site, lowered the affinity to Le^x^ (Fig. 4, Table 2). We also found that Le^x^ bound more weakly to CTB than Le^y^ or A-Le^y^, which is in good agreement with inhibitor studies identifying Le^y^ as the most potent small-molecule inhibitor of CTB^33^. The binding mode of Le^x^ is highly similar to that of Le^y^ tetraose and A-Le^y^ pentaose^21^, which both feature an additional fucose residue (Fig. 2c). The partial competition of GM1 and Le^x^ observed by Cervin *et al*.^32^ is most likely due to allosteric cross-talk, in line with recent findings^49,50^. Likewise, we found that the CT secondary binding site variant H18A exhibits increased GM1os binding.

### Comparison of ligand structures to structure–activity relationship data

l-fucose has been shown to block CTB binding in cell culture, serving as a promising starting point for the design of novel cholera inhibitors^33^. Recently, Wands *et al*. described important molecular determinants of l-fucose by competition binding assays of CTB to various intestinal epithelial cells^33^. Their structure–activity relationship (SAR) studies showed that the stereochemical positioning of the terminal fucose is crucial for CTB binding. While the removal of hydroxyl groups on C3 or C4 (OH3, OH4) had a significant effect on CTB binding, substitution of the hydroxyl group at C2 (OH2) did not result in reduced binding (compare Fig. 2c). The addition of a hydroxyl group to C6 was tolerated, but the removal of C6 resulted in a complete loss of CTB binding suggesting the importance of a hydrophobic patch within the binding pocket. Addition of a methyl group to OH1, locking the anomeric hydroxyl group in the β-configuration, resulted in less efficient CTB binding compared to l-fucose or α-locked fucose. Finally, the SAR study showed increased binding of α1,2-linked fucose compared to α1,3-linked fucose^33^.

All crystal structures of CTB with Le^x^ (6HJD; this work) and related molecules, such as Le^y^ or A-Le^y^ (5ELB^21^ and 5ELD^21^), show that fucose OH3 can form one or two hydrogen bonds to His94 and to a conserved water molecule (not shown in Fig. 2), explaining why removing OH3 in the SAR study caused reduced CTB binding. OH4 was reported to contribute the strongest to CTB binding^33^, which is in good agreement with the fact that OH4 forms two hydrogen bonds to backbone atoms of residues 47 and 94 (Fig. 2d). The crystal structures also identify a match for the proposed key hydrophobic residues interacting with C6 in Phe48, possibly together with Ala46 (Fig. 2d).

CTB binds l-fucose with its free anomeric hydroxyl group in the β-configuration (Fig. 3b), thus free fucose is not limited to bind in the α-configuration. However, locking the fucose in the β-configuration by the addition of a methyl group would cause steric clashes with residues Thr47 and Gly45, explaining why α-linked fucose bound stronger to CTB than β-linked fucose^33^.

According to Wands *et al*., OH2 does not contribute significantly as a hydrogen bond acceptor^33^, whereas we observe a hydrogen bond to Gln3# from the adjacent B-subunit (Fig. 2d). However, their conclusion was based on a compound with a fluorine replacing the OH2 group (rather than with OH2 removed), and it is still under debate if fluorine can form hydrogen bonds^51,52^.

Finally, Wands *et al*. found increased inhibition of CTB binding by fucosyllactose with α1,2-linked compared to α1,3-linked fucose residues^33^. The latter showed no difference compared to l-fucose alone. The crystal structure of CTB in complex with Le^y^ (5ELB^21^) clearly shows that both α1,2-linked and α1,3-linked fucose can bind to CT. However, the preferred orientation appears to be highly influenced by the precise details of ligands and toxins. For example, the structure of a CTB-LTB chimera in complex with a human milk oligosaccharide similar to A-Le^y^ exhibited exclusive binding of Fucα2 in the fucose binding pocket of the toxin^25^.

In conclusion, the structural data presented here are a very good match to the reported structure–activity relationship data, pointing to the same binding site identified in both studies, *i*.*e*. the secondary binding site of the toxin.

### Roles of GM1 and fucosylated receptors

A large body of research points to GM1 and fucosylated structures facilitating toxin binding and uptake^12,16–20,30,32–34^, however, little is known about the exact localization and expression of specific fucosylated structures on healthy intestinal cells and tissues^32,53–57^. It is not clear which fucosylated structures are the major cellular attachment sites besides GM1. However, all evidence points towards glycoproteins with type-2 core structures similar to Le^x^, Le^y^ and related HBGAs^21,27–30,32,33^. In particular, Le^x^ has been shown to allow CTB binding to intestinal cells^32^.

Whether the binding of GM1 and fucosylated structures happens simultaneously or sequentially is also unknown, nor what triggers or causes toxin uptake. Recent crystal structures from our lab suggest that CTB can bind sugars at both binding sites at the same time, specifically Le^y^ tetraose and galactose^21^. However, it has not yet been shown if this also holds true for GM1os. A recently published hetero-multivalent binding model suggests that CTB first binds to a high-affinity ligand such as GM1. Subsequent binding occurs much more readily (up to 10,000-fold faster) also for low-affinity ligands like GM2, since binding is then confined to the 2D membrane surface^58^. In fact, cooperativity was found to be enhanced for a heterogeneous mixture of ligands^58^. In a biological context, it seems more likely that the toxins first bind to the broadly available fucosylated structures on the cell surface or mucus layer until they can bind to the few available GM1 receptors, preventing the flow in the small intestine to remove unbound toxins from the intoxication site^21^. In addition, *V*. *cholerae* has other virulence factors that may influence receptor distribution and availability, *e*.*g*. neuraminidase VcN^59^. It has been shown recently that VcN can remodel intestinal polysialylated gangliosides into GM1 to provide an increased numbers of cellular receptors for CT^59^, and it is well conceivable that fucosylated glycoconjugates serve as intermediate anchoring points until VcN unveils enough GM1 receptors. Indeed, several studies showed that the exogenous addition of GM1 to cells markedly increases CT binding and intoxication^12,34,60,61^. Remarkably, a new study by Sethi *et al*. indicates that fucosylated glycoconjugates play a functional role in the process, in the presence or absence of GM1^34^.

### Correlation with epidemiological data: Why are secretors protected?

Many diseases show blood group association^62^, and cholera is the textbook example. Individuals with blood group O experience the most severe cholera symptoms^35–38^. Similarly, individuals with non-secretor phenotype are at increased risk of severe cholera, whereas so-called ‘secretors’, who secrete HBGAs and display these structures in their small intestinal mucosa, enjoy some protection^63,64^. Recently, progress has been made in the understanding of cholera ABO(H) blood group dependence^21^, however, it remains unclear why secretors are protected from severe cholera compared to non-secretors. The work presented here for Le^x^ represents the first relevant structural and affinity data for a non-secretor HBGA, bringing us in a unique position to shed light on this phenomenon.

Non-secretors have a non-functional *Se* (*FUT2*) gene, which codes for an α1,2-fucosyltransferase that is important for the generation of HBGAs on epithelial and endothelial cells and most body fluids^39^. Non-secretors therefore display a distinct set of receptors on their intestinal cells that is enriched in Lewis^a^ (Le^a^) and Le^x^ antigens, and characterized by limited fucosylation^39,55,65^. Interestingly, when probing jointly for secretor status and ABO(H) blood group, only secretors with blood group A or B showed less severe cholera symptoms, whereas the disease burden for blood group O individuals was equally high independent of secretor status^64^, which could be explained if the H-antigen characteristic of blood group O itself is a risk factor for severe cholera.

The H-antigen exists in two types, type-1 and type-2, with different linkages, both of which contain an α1,2-linked fucose. Addition of a second fucose residue (Fucα4 or Fucα3) then gives rise to Le^b/y^ determinants, respectively. If the same fucose is added to HBGA precursors instead, this yields Le^a/x^ epitopes, which in contrast to H-antigens and Le^b/y^ cannot be further modified by A or B glycosyltransferases, since they lack the α1,2-linked fucose. So far, all evidence points towards only type-2 antigens being able to bind to the CT^13,21,25^, focusing the attention on Le^y^ and Le^x^. The lower affinity of Le^x^ compared to the other HBGAs makes it unlikely that Le^x^ binding is the cause for more efficient CT uptake in non-secretors. There are only limited data regarding the expression of fucosyltransferases and the distribution of glycoconjugates in human tissues^32,53–56,66^. Moreover, it is unknown which fucosylated CT receptors are relevant *in vivo*. The available data, however, suggest that compared to the villi, the deeper parts of the intestinal membranes are unaffected by the non-functional FUT2 of non-secretors, due to expression of an alternative 1,2-fucosyltransferase encoded by the *H* gene^65,67^. Non-secretors cannot secrete soluble ABO(H) glycans, but might still present suitable docking sites (*i*.*e*., Le^y^) on these deeper membrane regions, independent of their secretor status, and cooperative binding is expected to be enhanced for hetero-multivalent binding including GM1^58^. Indeed, Cervin *et al*. observed tighter binding of CTB to the crypts than to the villi^32^. Due to the lack of soluble HBGAs, non-secretors would moreover lack competition by soluble and mucin-bound CT receptors, leading to more efficient CT uptake and more severe cholera symptoms.

### Conclusions and perspective

The importance of fucosylated sugars for cholera intoxication has recently been in the limelight^21,27–34^, but there has been limited structural information on their interaction with the CT. Here we show that fucosylated receptors, except fucosyl-GM1, bind solely to the secondary CT binding site, in contrast to recent suggestions^32^. We provide detailed information on how Le^x^ and l-fucose bind to CTB, and corroborate our findings with quantitative binding data of holotoxin variants. In addition, we present the first CT variant – H18A – with reduced affinity to fucosylated structures, but slightly increased affinity to GM1os. This not only lends further support to a possible cross-talk between the primary and secondary CT binding sites, but may also have practical value. Currently CT is widely used as a marker of GM1 and lipid rafts^68^. We suggest to replace wild-type CTB with variant H18A as more specific GM1 marker, to limit the number of false-positive results caused by the interaction with secondary binding site receptors. Furthermore, CT variants H18A and H18AH94A, being devoid of a functional secondary binding site, are predicted to facilitate the analysis of cellular uptake in cellular and organoid models^31,69^ by allowing discrimination of the effects caused by interaction with primary and secondary sites.

## Materials and Methods

### Mutagenesis

Vector pARCT5, which was kindly provided by Professor Randall K. Holmes, contains an arabinose-inducible CT operon, with signal sequences derived from the LT-IIb B gene^70^. Site-directed mutagenesis was performed using the manufacturer’s protocol (Q5® Site-Directed Mutagenesis Kit, NEB) and DNA oligo nucleotides (Eurofins Genomics) shown in Table 3. Successful mutagenesis was verified by Sanger sequencing using primers Seq 1–3 (Eurofins Genomics, GATC).

**Table 3:** DNA oligonucleotides used in this study.

DNA oligo name	DNA sequence (changed bases in bold)
W88K_Fwd	GTTATGTGTA**AAG**AATAATAAAACGCCTC
W88K_Rev	TTTTCGACTTTAGCTTCAG
H18A_Fwd	CACACAAATA**GCG**ACGCTAAATGATAAGATATTTTC
H18A_Rev	TTGTGGTATTCTGCACAC
H94A_Fwd	TAAAACGCCT**GCG**GCGATTGCCGC
H94A_Rev	TTATTCCATACACATAACTTTTC
Seq 1	GAT CTT GGA GCA TTC CCA CA
Seq 2	TTA TAG CCA CTG CAC CCA ACA TG
Seq 3	CAA GAG ATT ACG CGC AGA CC

### Production of classical CTB, standard protocol

Expression was performed essentially as described previously^21^. Briefly, the gene for CTB (Uniprot: Q57193; classical biotype) was heterologously expressed in *E*. *coli* BL21 (DE3) using a CTB-pET21b+ construct. For protein production, cells were grown at 37 °C in LB medium containing ampicillin until OD_600 nm_ of 0.5 was reached. The temperature was reduced to 25 °C and isopropyl-β-d-thiogalactopyranoside (IPTG) was added to a final concentration of 0.5 mM to start CTB production. Cells were harvested after 14–18 h by centrifugation (6900 × *g*, 20 min, 4 °C) and the pellet was re-suspended in ice-cold sucrose buffer (20 mM Tris/HCl, 25% (w/v) sucrose, 5 mM EDTA at pH 8.0). After 15 min on ice, the solution was centrifuged (8000 × *g*, 20 min, 4 °C) and the pellet was re-suspended in periplasmic lysis buffer (5 mM MgCl_2_, 150 μg/mL lysozyme, DNase). The solution was kept cold for 30 min, centrifuged (8000 × *g*, 20 min, 4 °C) and dialyzed for several hours against PBS in a Snakeskin tube (Thermo Scientific, 3500 MWCO).

### Production of CTB and CT holotoxins for TALON chromatography

The gene for CTB (Uniprot: Q57193) was heterologously expressed in *E*. *coli* BL21 (DE3) using a CTB-pET21b +construct. For protein production, cells were grown at 37 °C in LB medium containing ampicillin until OD_600 nm_ of 0.5 was reached. The temperature was reduced to 25 °C and IPTG was added to a final concentration of 0.5 mM to start CTB production.

The genes for CT and CT variants (W88K, H18A, H18AH94A) were heterologously expressed in OverExpress™ C43 (DE3) cells (Sigma) using pARCT5 or pARCT5 derivatives. For protein production, cells were grown at 37 °C in TB medium containing chloramphenicol until OD_600 nm_ of 2.0 was reached. l-arabinose was added to a final concentration of 0.2% (w/v) to start holotoxin production.

Cells were harvested after 14–18 h (CTB) or 3 h (holotoxin) by centrifugation (6900 × *g*, 20 min, 4 °C) and the pellet was re-suspended in 1/60^th^ volume of TALON A buffer (50 mM sodium phosphate, 300 mM NaCl, pH 8) with 1 mg of polymyxin B (Sigma Aldrich) per mL, cOmplete™ Protease Inhibitor (Roche) and benzonase (EMD Millipore) and shaken at 37 °C for 15 min. Insoluble debris and cells were removed from the periplasmic extracts by centrifugation (8000 × *g*, 20 min, 4 °C). The filtered supernatant was directly applied to the TALON affinity column.

### Purification of CTB using D-galactose affinity chromatography

The protein solution was loaded onto a d-galactose-sepharose affinity column (Thermo Scientific) and eluted using 300 mM galactose in PBS. Fractions containing pure CTB were pooled and concentrated using Vivaspin 20 mL concentrator tubes (5000 MWCO, PES membrane, Sartorius). The protein was subjected to size-exclusion chromatography using PBS and a Superdex 75 column mounted on an ÄKTA purifier (GE Healthcare). Fractions with toxin were pooled, dialyzed over night against Tris buffer (20 mM Tris/HCl, 200 mM NaCl, pH 7.5), concentrated to 3–9 mg/mL, snap-frozen in liquid nitrogen and stored at −80 °C.

### Purification of CTB and CT holotoxins using TALON affinity chromatography

As alternative to the d-galactose-sepharose affinity purification (to preclude contamination with galactose), the periplasmic extract of CTB or CT (wild-type and variants of classical biotype) was loaded onto a pre-equilibrated HiTrap TALON crude column (GE Healthcare) and eluted with TALON B buffer (50 mM sodium phosphate, 300 mM NaCl, 50 mM imidazole at pH 8) in order to avoid residual D-galactose bound to the protein. The protein solution was concentrated using Vivaspin concentrator tubes (10000 MWCO, PES membrane, Sartorius) and loaded onto a Superdex 75 (CTB) or Superdex 200 column (holotoxins) mounted on an ÄKTA purifier (GE Healthcare) equilibrated with Tris buffer (20 mM Tris/HCl, 200 mM NaCl at pH 7.5) or PBS. Fractions with CTB pentamer (Tris buffer) were pooled, concentrated to 3 mg/mL, snap-frozen in liquid nitrogen and stored at −80 °C. Fractions with holotoxins (PBS) were pooled and stored at 4 °C.

### CD spectroscopy and MS

Correct folding of all CT variants was verified by CD spectroscopy (J-810 CD spectrometer, JASCO). Prior to the measurement, buffer exchange to 10 mM sodium phosphate buffer pH 7.5 was performed using Vivaspin concentrator tubes. All CT variants were characterized by SDS-PAGE analysis, tryptic digestion and mass spectrometry (performed in house). The amino acid substitution H94A was only confirmed indirectly by DNA sequencing, since tryptic digestion and MS did not yield a suitable C-terminal peptide.

### Co-crystallization of CTB complexes

CTB (Gal-affinity) and Le^x^ triaose (Gly049, Elicityl) or CTB (TALON) and fucosyl-GM1os (GLY103, Elicityl) were mixed at a molar ratio of 1:10 (B-subunit to ligand). CTB (TALON) and l-fucose (F2252, Sigma Aldrich) were mixed at a molar ratio of 1:500 (B-subunit to ligand), corresponding to a final concentration of 0.14 M of l-fucose, for a protein concentration of 3.3 mg/ml. All samples were incubated at RT for 2 h. Initially, crystals were obtained by sitting-drop vapour-diffusion using a crystallization robot (Oryx4, Douglas Instruments) at 20 °C in the Morpheus screen^71^ condition A12 (Molecular Dimensions). For CTB and Le^x^ triaose another crystal form was obtained in Morpheus screen condition A4. Crystals from condition A12 were optimized for all ligands by several rounds of hanging-drop vapour-diffusion experiments. Final crystals for the CTB complex with Le^x^ were obtained by mixing protein-ligand solution (1 µL, 3.3 mg/mL) 1∶1 with crystallization buffer (0.1 M Tris (base)/BICINE pH 8.5, 8% PEG1000, 8% PEG3350, 8% MPD, 0.03 M MgCl_2,_ 0.03 M CaCl_2_) and 0.2 µL seed solution (microseeding, Seed bead kit, Hampton Research). CTB l fucose co-crystals were obtained by mixing protein-ligand solution (1 µL, 3 mg/mL) 1:1 with crystallization buffer (0.1 M Tris (base)/BICINE pH 8.7, 10% PEG1000, 10% PEG3350, 10% MPD, 0.03 M MgCl_2_, 0.03 M CaCl_2_). Final crystals for the CTB complex with fucosyl-GM1os were obtained by mixing protein-ligand solution (1.5 µL, 1.5 mg/mL) 1:1 with crystallization buffer (0.1 M Tris (base)/BICINE at pH 8.7, 6% PEG1000, 6% PEG3350, 6% MPD, 0.03 M MgCl_2_, 0.03 M CaCl_2_) and cryoprotection was achieved by transferring this crystal to crystallization buffer with higher PEG and MPD concentrations (0.1 M Tris (base)/BICINE pH 8.7, 10% PEG1000, 10% PEG3350, 10% MPD, 0.03 M MgCl_2_, 0.03 M CaCl_2_. Crystals were harvested, mounted in loops, and flash-cooled in liquid nitrogen (CTB l fucose, CTB fucosyl GM1os) or a nitrogen cryo-stream (CTB-Le^x^), respectively.

### X-ray data collection and refinement

Synchrotron data collection for the CTB complex with Le^x^ was performed at ID23–1, ESRF, Grenoble, France (100 K, 0.9770 Å). Data collection for CTB complexes with l-fucose or fucosyl-GM1os was performed at BioMAX, Max IV, Lund, Sweden (100 K, 0.9795 Å and 0.9808 Å, respectively). Data were processed with *XDS*^72^ and NEGGIA/DECTRIS (CTB l fucose, CTB fucosyl GM1os) or *xia2* and *DIALS* (CTB-Le^x^)^73,74^ and *AIMLESS* from the *CCP*4 software suite^75,76 (Table 1)^. Diffraction data cut-offs were chosen based on the assessment of the anisotropic *CC*_1/2_ and the quality of the electron density. Both crystal forms of CTB-Le^x^ belong to space group *P*2_1_2_1_2_1_ and contain two B-pentamers in the asymmetric unit, but have different cell parameters. For the CTB-Le^x^ model, we used the highest resolution data set (1.5 Å) from an optimized Morpheus A12 condition, which crystallized under similar conditions, and with the same space group and pentamer arrangement (“top-to-top”) as the CTB complex with Le^y^ tetraose reported previously (5ELB^21^). CTB l fucose and CTB fucosyl GM1os crystals belong to space group *C2* and contain two B-pentamers in the asymmetric unit. The structures were solved by molecular replacement using *Phaser*^77^ from the *CCP*4 software suite^75,76^ and search model 5ELB^21^, from which ligands and water molecules had been manually removed. To avoid potential model bias, five cycles of refinement including two cycles with simulated annealing (starting temperature of 5000 K) were carried out with the Phenix software suite^78^. The final model was obtained after several cycles of manual building with *Coot*^79^, followed by refinement with *REFMAC5*^80^. Initial refinement steps involved local NCS restraints, while final refinement steps involved TLS parameterization (*REFMAC5*, automatic, 5 cycles)^81^. Water molecules were placed using COOT:Find_waters and then manually inspected for several criteria, including distances from hydrogen-bond donors/acceptors and quality of the electron-density. Most of the disulfide bridges are partly reduced, due to minor radiation damage. Le^x^ triaose, GM1os and fucosyl-GM1os were built using MAKE LIGAND (*AceDRG*)^82^ from the *CCP*4 software suite^75,76^ and isomeric SMILES strings. The restraints for a Thr-Fuc bond were generated using *JLigand*^83^. Le^x^ triaose, fucosyl-GM1os and α-l-fucose or β-l-fucose were included last to avoid model bias. To improve the density for the terminal fucose residue, GM1os was included prior to fucosyl-GM1os. For the CTB complex with fucose, additional elongated electron density was found in two of the primary binding sites, however, the origin of the density could not be identified, even with Polder^84^ maps calculated with the Phenix software suite^78^ (the density was clearly not compatible with a sugar ring). PDB_REDO^85^ was used to evaluate the models before final refinement steps. Occupancies were refined by evaluating the difference Fourier maps and by comparing the *B*-factors of the ligands with interacting protein atoms (exception: fucose residues in the CTB-fucosyl-GM1os secondary sites, which were modelled at full occupancy). The final models were analysed using the Analyse geometry task of the *CCP*4 software suite^75,76^. The percentages of amino acid residues occupying the favoured, allowed and outlier regions in the Ramachandran plot are 97.5/2.5/0.0% for CTB-Le^x^, 97.4/2.4/0.2% for CTB l fucose, and 97.7/2.3/0.0% for CTB-fucosyl-GM1os, respectively. Figures were generated using PyMol (Schrödinger LLC), α-helices and β-strands were assigned using STRIDE^86^.

### Surface plasmon resonance spectroscopy

SPR analyses were performed using Series S CM5 sensor chips and a Biacore T100 biosensor system (Biacore Life Sciences, GE Healthcare). Due to the high cost of the oligosaccharides combined with the relatively low affinity typical for carbohydrate-protein interactions, experiments were performed in duplicates or triplicates. Proteins were immobilized on the surface of the sensor chip to yield a signal of approximately 4200 response units, as described previously^21^. Le^x^ tetraose (GLY050, Elicityl) and triaose were dissolved in the running buffer (10 mM HEPES/NaOH, 140 mM NaCl, 3 mM EDTA, 0.005% P20, pH 7.4) and injected in concentrations from 78.125 μM up to 40 mM for 45 s. Dissociation was monitored for additional 15 s. The regeneration was not needed for Le^x^ tetraose, whereas for Le^x^ triaose, a short pulse (6 s) of 3 mM NaOH was injected to completely remove it from the surface. Binding of 3.125–200 nM GM1os (GM1a, GLY096, Elicityl) was monitored for 240 s, with additional 420 s for dissociation. The regeneration of the chip was the same as for Le^x^ triaose. All binding steps were performed at 30 μL/min and 25 °C. The data were evaluated using Biacore T100 Evaluation software. For GM1os, data were fitted to the Langmuir 1:1 interaction model, since responses did not reach the equilibrium during the association phase. Other binding affinities were determined from plots of steady-state analyte binding levels against the concentration.

## Supplementary information

Supplementary Information

## Data Availability

The coordinates and structure factors have been deposited with the Protein Data Bank with accession codes: 6HJD, 6HMW and 6HMY.
