# Supplementary material for: Crystal structures of cholera toxin in complex with fucosylated receptors point to importance of secondary binding site
Source: Sci Rep. 2019 Aug 22;9:12243. doi: 10.1038/s41598-019-48579-2 (PMC6706398; doi:10.1038/s41598-019-48579-2)
Supplement: Supplementary file 1 — Supplementary Information [file 41598_2019_48579_MOESM1_ESM.pdf]

Supplementary information

Crystal structures of cholera toxin in complex with fucosylated receptors point to importance of secondary binding site

Joel B. Heim[1], Vesna Hodnik[2,3,4], Julie E. Heggelund[1], Gregor Anderluh[3], Ute Krengel[1*]

[1] Department of Chemistry, University of Oslo, P.O. Box 1033, NO-0315, Blindern, Norway

[2] Department of Biology, Biotechnical Faculty, University of Ljubljana, Jamnikarjeva 101, 1000 Ljubljana, Slovenia

[3] Department of Molecular Biology and Nanobiotechnology, The National Institute of Chemistry, Hajdrihova 19, 1000 Ljubljana, Slovenia

[4] Present address: Lek d.d., Kolodvorska 27, 1234 Mengeš, Slovenia

[*]Corresponding author

E-mail: ute.krengel@kjemi.uio.no

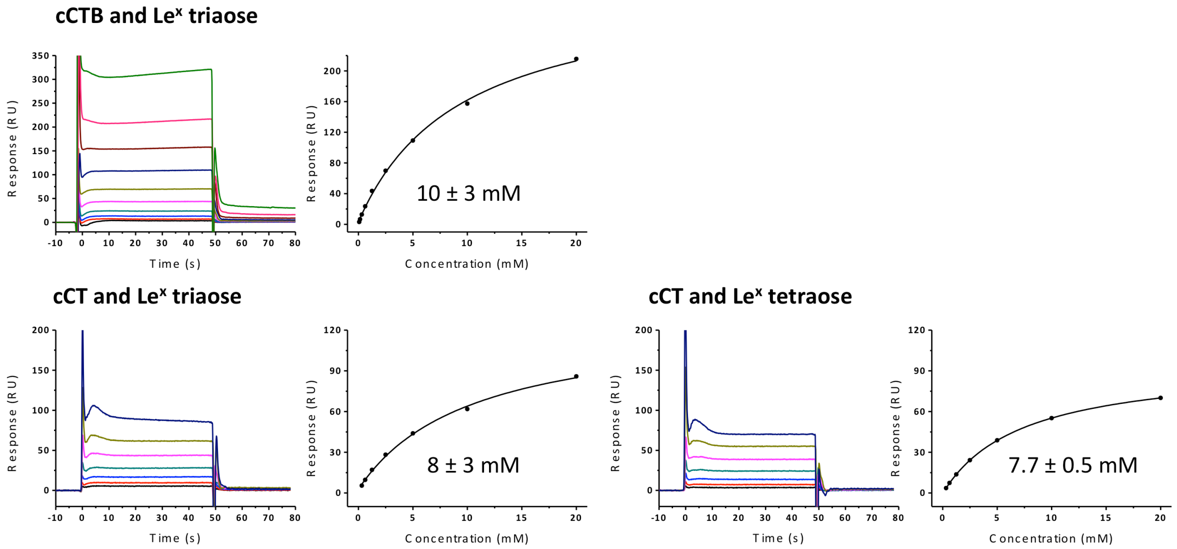

Figure S1. SPR sensorgrams and affinity plots for cholera toxin B pentamer and cholera holotoxin. SPR experiments were performed with cCT B pentamers or holotoxins coupled to the sensor chip and using Le^{x} triaose or tetraose as analytes, as indicated in the panel legends (sensorgrams and corresponding plots of steady state response against concentration).

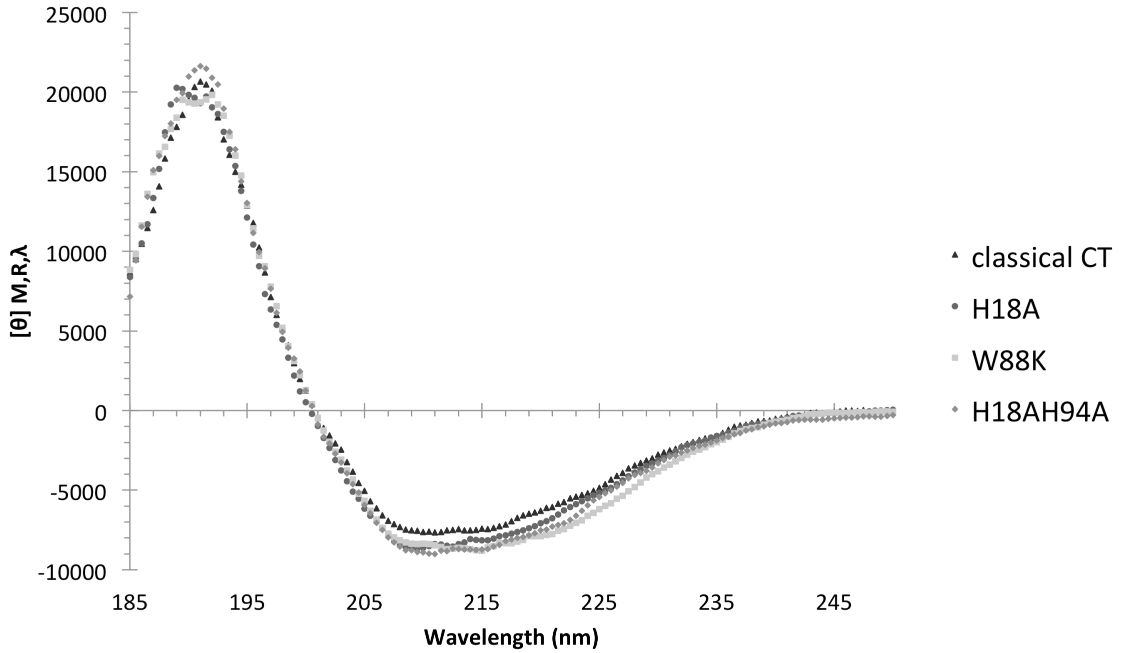

Figure S2. Assessment of the folding of CT variants by circular dichroism (CD) spectroscopy. CD spectra of recombinant wild-type cCT holotoxin (triangles) and cCT variants H18A (dots), W88K (squares) and H18AH94A (diamonds). Results indicate folded proteins with similar folds.

Table S1. Protein-carbohydrate interactions in cCTB-Le^{x}

Ligand residue	Direct contact (Å)	Interaction partner
Fuc O4	2.9 ± 0.1(8/10)	His94 N
Fuc O4	2.6 ± 0.1 (9/10)	Thr47 O
Fuc O5	3.0 ± 0.0 (9/10)	Thr47 N
Fuc O3	3.0 ± 0.3 (4/10)	W1
Fuc O2	2.7 ± 0.1 (9/10)	Gln3# Oε/Nε
GlcNAc N2	2.9 ± 0.1 (8/10)	Gly45 O
GlcNAc O1	3.1 ± 0.2 (6/10)	Asn44 O
Gal O4	2.9 ± 0.1 (3/10)	His 4 Nε

Listed are polar interactions of max 3.5 Å and with favourable angles for H-bonds. The number of binding sites, in which the interaction is present, is shown in parentheses. Residues from adjacent subunits are labelled with a hash (#).

Table S2. Protein-carbohydrate interactions in cCTB-L-fucose

Ligand residue	Direct contact (Å)	Interaction partner
Fuc O4	2.9 ± 0.1 (9/10)	His94 N
Fuc O4	2.7 ± 0.1 (9/10)	Thr47 O
Fuc O5	3.1 ± 0.1 (9/10)	Thr47 N
Fuc O3	3.4 (1/10)	W1
Fuc O2	2.6 ± 0.1 (9/10)	Gln3# Oε/Nε
Fuc O2	3.4 (1/10)	W1
Fuc O1	3.2 ± 0.1 (7/10)	Thr47 N
Fuc O1	3.0 ± 0.1 (9/10)	Gly45 O
Fuc O1	3.1 ± 0.3 (7/10)	Thr47 Oγ1

Listed are polar interactions of max 3.5 Å and with favourable angles for H-bonds. The number of binding sites, in which the interaction is present, is shown in parentheses. Residues from adjacent subunits are labelled with a hash (#).
